# Improving care for women living with HIV: initial outcomes of an integration experience

**DOI:** 10.7448/IAS.15.6.18088

**Published:** 2012-11-11

**Authors:** V Fink, D Zurita, M Tejo, H Perez, C Cesar, M Figueroa, P Patterson, O Sued, P Cahn

**Affiliations:** 1 Hospital Juan A Fernandez, Infectious Diseases Unit, Buenos Aires, Argentina; 2 Hospital Juan A Fernandez, Department of Gynecology, Buenos Aires, Argentina; 3 Hospital Juan A Fernandez, Department of Pathology, Buenos Aires, Argentina

## Abstract

**Background:**

Women living with HIV are at higher risk of developing HPV-related diseases. Albeit they are systematically referred for cervical cancer screening, difficulties in obtaining timely appointments are the main barrier for an adequate gynecological care. In our unit, according to a previous survey, 67% of women reported this problem. Therefore, in January 2011 the integration of HIV and gynecological care was sought through the provision of gynecological care within the Infectious Diseases Unit in our hospital.

**Methods:**

A weekly specific clinic for women living with HIV cared by HIV and gynecological specialists was implemented. Appointments are given at the HIV clinic, with no need of referral. Pap smear and colposcopy are offered in the same place. Data are collected through standardized forms. Baseline data from the first hundred patients referred are presented.

**Results:**

Ninety-six women were assisted. Median age was 40 years (IQR 36–46.5). Median time from HIV diagnosis was 10.6 years (IQR 4.9–16.4). 82% patients were on HAART. Median CD4 cell count was 473 cells/cc (IQR: 287–614) and 49% had viral load<50. 48% lacked a gynecological control for the last 2 years. 24% had been previously diagnosed and/or treated for HPV-related pathology. Cervical Pap smear results (n=94): 59% were negative; 20% had LGSIL and 2% had HGSIL. Of those diagnosed with SIL, 29% had history of HPV-related lesions. Of note, 23% had infections or inflammatory results. Clinically significant abnormal colposcopies were seen in 21/93 (23%) patients. Of those, 30% were diagnosed SIL in the Pap smear.

**Conclusions:**

Integrating the gynecologist with the ID Unit allowed women living with HIV easier access to gynecological control. The high number of abnormalities in the Pap smears detected in this pilot study reinforces the need of improving cervical cancer screening for prevention and early treatment through integrated approaches.

